# Risk Factors of Dental Caries in Preschool Children in Thailand: A Cross-Sectional Study

**DOI:** 10.3390/healthcare10050794

**Published:** 2022-04-25

**Authors:** Manarin Boonyawong, Prim Auychai, Duangporn Duangthip

**Affiliations:** 1 Department of Pediatric Dentistry, Faculty of Dentistry, Chulalongkorn University, Bangkok 10330, Thailand; bess.manarin@gmail.com; 2 Faculty of Dentistry, The University of Hong Kong, Hong Kong; dduang@hku.hk

**Keywords:** dental caries, risk factor, preschool, Thailand

## Abstract

Dental caries remains prevalent in young children. This study determined dental caries prevalence and risk factors associated with caries experience in Thai preschool children. Five kindergartens in Samut Sakhon Province were evaluated. Preschool children (4- to 5-year-old children) were recruited. The participants’ parents completed a questionnaire regarding their children’s demographic and socio-economic backgrounds and their oral health-related behaviors. Dental caries status and oral hygiene were recorded using the decayed, missing, and filled teeth index (dmft) and visible plaque index (VPI), respectively. In total, 308 children completed the oral examination (93.9% response rate). The mean age of the children was 5.1 ± 0.5 years old. Among them, 249 children (80.8%) had dental caries (dmft > 0) and their mean dmft score (SD) was 8.2 (4.7). The children’s age, VPI, primary caregiver, age of starting tooth brushing, assisted tooth brushing, and mother’s education level were significantly associated with dental caries (X^2^test, *p* < 0.05). The multiple logistic regression analysis revealed that older children with a higher VPI score and whose mothers had lower education had a significantly higher risk of having dental caries (*p* < 0.05). Caries prevalence was high among the evaluated Thai preschool children. The child’s age, visible dental plaque, and mother’s educational level are significant risk factors for dental caries.

## 1. Introduction

Dental caries is a common oral disease affecting children worldwide [1,2]. In Thailand, the results of the National Oral Health Survey conducted in 2017 indicated that more than half (53%) of 3-year-old children and 76% of 5-year-old children have a caries experience [3]. Dental caries is a multifactorial disease that is affected by cariogenic plaque, fermentable carbohydrates, a susceptible tooth, and time, as well as environmental factors, such as saliva, availability of fluoride, parental dental knowledge, access to dental care, and socioeconomic background [4,5,6,7]. Previous studies found that dietary habits (e.g., sugary snacks), poor oral hygiene, microbiological factors, and low economic status were significantly related to dental caries [8,9]. 

The prevalence of dental caries is substantially higher among poorer and disadvantaged populations in developed and developing countries [2,10,11]. A review also reported the developing dental caries was strongly associated with social, political, cultural, and educational conditions, which influence personal and collective hygiene habits and consequently interact with the biological determinants of the disease [12]. Studies have revealed that living conditions that reflect the socio-economic status and residential location can influence access to health resources [13]. 

Urbanization is a complex socio-economic process that transforms the built environment, converting formerly rural areas into an urban setting, while also shifting the spatial distribution of a population from rural to urban areas. It includes changes in the dominant occupations, lifestyle, culture, and behavior of the population, and thus alters the demographic and social structure of the urbanized areas [13]. Currently, the ongoing urbanization in Thailand offers the rural population opportunities for economic improvement and lifestyle changes. However, there are no studies on the caries prevalence in areas undergoing urbanization in Thailand. The Ban Phaeo District, Samut Sakhon Province, which is a semi-urban area, is an example where people’s lifestyle has rapidly transformed from rural to urban. Samut Sakhon is one of the central provinces and it is a part of the Bangkok Metropolitan Region. Previously an agricultural- and fisheries-based area, Samut Sakhon has changed to establish thousands of small factories in these recent years. The majority of the residents work as fishers, farmers, and factory workers. Fluoride in drinking water is between 0.5 and 0.7 ppm or above in the province [14]. As the caries prevalence in this semi-urban area and its associated risk factors are not known, understanding these can allow for the implementation of appropriate preventive methods to reduce the dental caries burden and improve children’s oral health.

Thus, the aim of this study was to determine the caries prevalence and its associated factors in preschool children in Samut Sakhon Province, Thailand.

## 2. Materials and Methods

This cross-sectional study was conducted from February to August 2019 in Samut Sakhon Province. The study protocol was approved by the Human Research Ethics Committee of the Faculty of Dentistry, Chulalongkorn University (HREC-DCU 2020-018). 

### 2.1. Sample Size Calculation 

The sample size estimation was based on the previous caries prevalence (~75%) [3]. The confidence interval was set at 5% (Cl: 70–80%) with a 95% confidence level. The estimated sample size was 289. With an estimated response rate of 90%, the number of children invited to take part in the study would be at least 321.

### 2.2. Recruitment of Participants

A quota sampling method was used. Five kindergartens in Ban Phaeo District, Samut Sakhon Province were invited to join this study. An invitation letter explaining the purposes and procedures of the study was sent to the parents. Written parental consent forms were obtained before the clinical examination were performed. The inclusion criteria of this study were generally healthy 4- to 5-year-old children. Children who were uncooperative or had significant systemic diseases were excluded.

### 2.3. Questionnaire Survey

Self-completed questionnaires and parental consent forms were distributed to the parents of children in the selected schools. Informed consent forms and questionnaires were collected before the clinical examinations. The parents were asked to complete a questionnaire with three sections: (i) demographic background: sex, age, and main caregiver; (ii) socio-economic status: parents’ education levels and family income; (iii) oral health-related habits: snacking habits and tooth brushing habits. Returned questionnaires with missing and incomplete answers were followed up by telephone.

### 2.4. Clinical Examination

A dentist (MB) who had been trained by a specialist in pedodontics (PA) and a specialist in dental public health (DD) performed the clinical examination in a kindergarten classroom using a dental explorer, a mouth mirror, and an electric light bulb. The caries diagnostic criteria followed the WHO recommendations [15]. Caries experience was recorded by using the dmft index. A tooth was recorded as decayed (dt) when a lesion had a visible cavity, undermined enamel, or soft dentin floor or wall. A tooth was recorded as missing (mt) when it was extracted due to caries. A tooth was recorded as filled (ft) when it was permanently filled without caries. The children’s oral hygiene was evaluated using visible plaque index (VPI) [16]. The visible plaque of buccal and lingual surfaces of six index teeth (55, 51, 63, 75, 71, and 83) were recorded as the presence of visible plaque (score 1) or the absence of visible plaque (score 0). The VPI score was calculated as the percentage of the number of surfaces with visible plaque relative to the total number of surfaces examine [17]. 

The intra-examiner agreement was assessed by re-examining a 20% random sample of children on a different day in the same week. A dental assistant selected the children to be re-examined. After the dental examination, each child received an oral health report. As stated in the parent information sheet approved by the Human Research Ethics Committee of the Faculty of Dentistry, Chulalongkorn University, no treatment was provided on-site. The parents could seek the appropriate dental treatment at their own cost.

### 2.5. Data Entry

The data were entered into a personal computer twice by two independent investigators. The two sets of data were compared using the Excel program. If discrepancies were found, the data were rechecked with the hard copy and revised accordingly.

### 2.6. Statistical Analysis

Data analysis was performed using Statistical Package for Social Science version 22.0 (SPSS Inc., Chicago, IL, USA). The intra-examiner agreement was assessed using Kappa statistics. Children were divided into two groups: no caries experience (dmft = 0) and caries experience (dmft > 0). The Chi-square test was used to investigate the association between the independent categorical variables evaluated and caries experience. The independent t-test was used to analyze the association between the independent variables (ratio scale) and caries experience. Multiple logistic regression analysis was used to study all potential variables related to dental caries experience. The statistical significance level for all tests was set at 0.05.

## 3. Results

**Figure 1:**
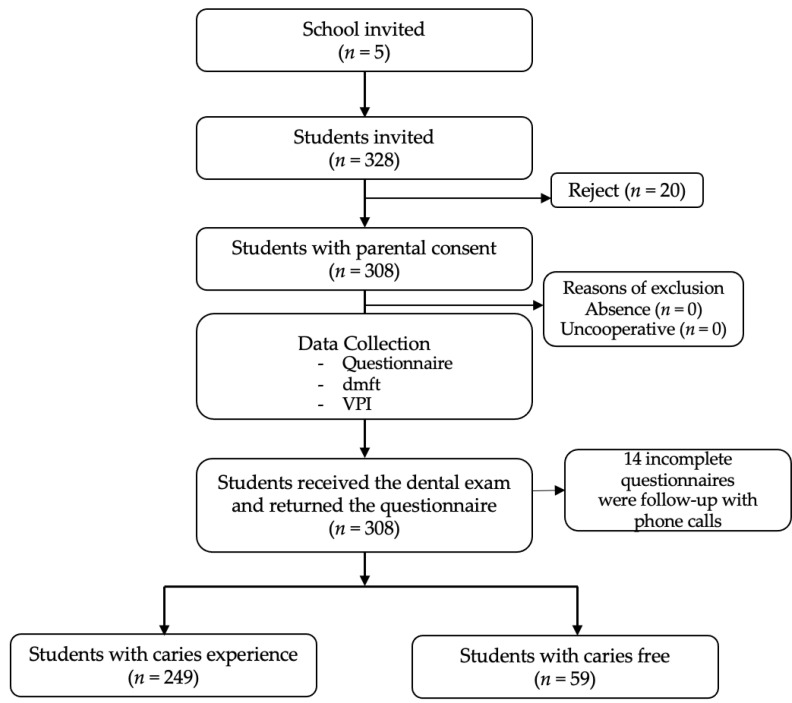
Research flow chart.

In total, 338 preschool children attending five kindergartens were recruited for this study. Parental consent forms and questionnaires were distributed. The final participants comprised 308 children (94.1% response rate) (Figure 1). The intra-examiner agreement (Kappa value) was 0.98. All 308 children with parental consent were clinically examined. Missing answers on the returned questionnaire were followed up by telephone. 

**Figure 2:**
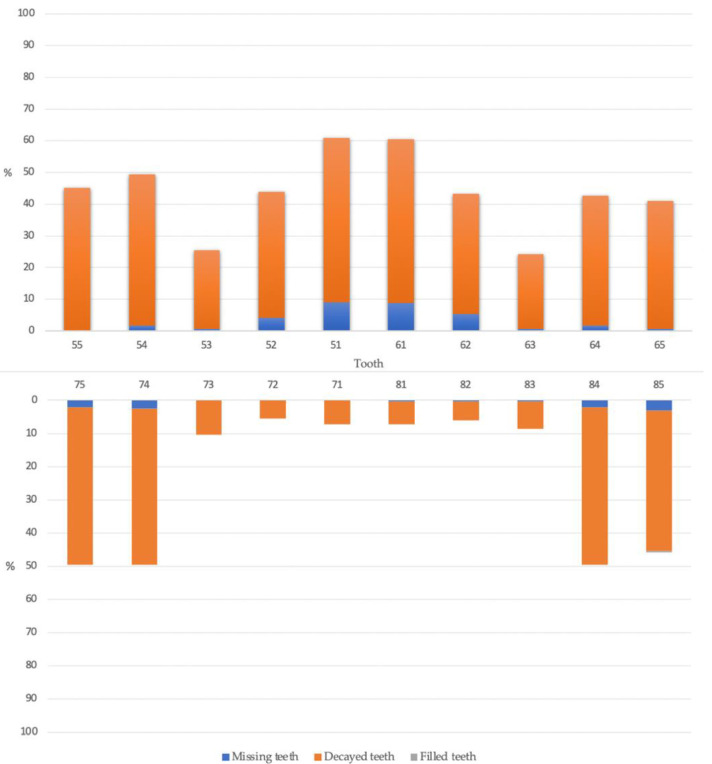
Distribution of caries experience (%) in each tooth in the maxillary and mandibular arch.

Among the 308 children, 249 (80.8%) children had caries experience (dmft > 0). The caries prevalence in 4- and 5-year-old children was 76.3% and 84.6%, and their mean dmft was 7.9 ± 4.2 and 8.9 ± 5.2, respectively. Among the study children, 156 (52.5%) were boys, and the mean age (±SD) was 5.1 ± 0.5 years old. There were no significant differences in the prevalence of caries between boys and girls. The number of children aged 4 and 5 years old in the study was 139 (45.1%) and 169 (54.9%), respectively. Most of the children had untreated caries with a mean ft (±SD) <0.1 (Table 1). The clinical examination results revealed that the mean dmft score of the caries experience group was 8.3 ± 4.7. Figure 2 shows the proportion of decayed, missing, and filled teeth in each tooth position to the number of all examined teeth in each tooth position. The *x*-axis is the position of primary teeth in the upper and lower arch. The *y*-axis is the percentage of decayed (orange), missing (blue), and filled (grey) teeth of the examined teeth in each tooth position. Maxillary central incisors had the highest prevalence of caries (60.7%), whereas mandibular central and lateral incisors had the lowest prevalence of caries (7.3% and 5.7%, respectively).

The associations between the caries experience and children’s demographic background and dental plaque are presented in Table 2. The child’s age, primary caregiver, mother’s education, age of starting tooth brushing, assisted tooth brushing, and visible plaque index were significantly associated with children’s dental caries experience (*p* < 0.05). Table 3 presents the oral health-related behaviors of children with and without caries experience. There was a significant association between caries experience and age of starting tooth brushing and assisted tooth brushing (*p* = 0.040 and 0.043, respectively). In contrast, there was no difference between caries experience and the child’s gender, father’s education, family’s income, age of starting using fluoride toothpaste, use of fluoride toothpaste, frequency of tooth brushing, eating after brushing at nighttime, or frequency of snacking (chi-square test, *p* > 0.05). The final model of the multiple logistic regression analysis revealed that the child’s age, visible plaque index, and mother’s education level were significant risk factors associated with dental caries (Table 4). Children with increased age had a significantly higher chance of having caries experience (2.4-fold). Children with high and moderate VPI index had a significantly higher chance of having caries experience (10.3- and 6.3-fold, respectively) compared with children with low VPI index. Children whose mother had a mandatory education had a significantly higher chance of having caries experience than children whose mother had a higher education (2.0-fold).

## 4. Discussion

The present study determined the caries prevalence and its associated factors in preschool children in Samut Sakhon Province, Thailand. The results demonstrated that the overall caries prevalence in 4- to 5-year-old children was very high. The dental caries prevalence in 5-year-old children in Ban Phaeo was 84.6%, which was higher than the national oral health report in 2017 (75.6%) [3]. Different environments, lifestyles, economic levels, dental health education, and family backgrounds may contribute to these differences. The results of the systematic review among 5- to 6-year-old children in Southeast Asia indicated that the caries experience is more widespread within lower socioeconomic status populations [17]. Similar to the results of the current study, the mother’s education level was a significant factor affecting children’s caries experience. Children with a lower mother’s education level had a higher chance to develop caries, compared with those with a higher mother’s education level. Mothers with higher education levels may also have higher dental knowledge levels, which might explain the better oral health of their children. Similar to the results the previous studies [17,18,19], the mother’s education level was also found to be related to children’s caries status. Mothers play a vital role in their children’s oral health in the early years of life. The effectiveness of oral health programs can be increased when the programs are tailored to increase the dental awareness and attitudes of targeted mothers who obtain a middle school or lower education level. 

In addition to the mother’s education, the dental caries prevalence was associated with the presence of visible dental plaque and the children’s age. Children with a moderate and high VPI index had a significantly higher caries risk compared with children with a low VPI index. The results of the current study agree with those of a previous study in infants and toddlers in Thailand [20]. Similarly, previous published studies in many countries concluded that visible dental plaque was the most important oral hygiene factor related to dental caries [18,21,22]. Oral health-related behaviors including age of starting tooth brushing and assisted tooth brushing were found to be significantly associated with children’s caries prevalence in the bivariate analysis. However, the final model after adjusting for confounders revealed that only VPI, age, and mother’s educational attainment were the significant factors. This may be the fact that dental plaque is the confounder affecting caries prevalence and it may be correlated with the age of starting tooth brushing and assisted tooth brushing.

The present study found an association between age and dental caries. This indicates that caries severity gradually increased with age in the primary dentition. Our results were consistent with those of several previous studies [10,18,21]. Because dental caries is a cumulative process, health promotion and dental prevention should begin as early as the first tooth erupts at the first year of their life [23]. A study has reported that the onset of caries is more likely when the children start brushing at a later age [24]. Parental indulgence (when parents neglected to help the child brush twice daily) was reported as one of the most important factors for dental caries [25]. Primary caregivers are the important key persons who are responsible for the initiation of tooth brushing or even assisted tooth brushing. Therefore, oral health education should be provided to primary caregivers. Families and nurseries/kindergartens should help children develop good oral hygiene and eating habits. Early intervention programs for preschool children’s oral health behavior should be developed based on the risk factors identified in this study. More importantly, policymakers should work hand in hand to improve the quality and accessibility of oral health services.

The strengths of the study included a high response rate (100%) of the recruited children. In addition, all incomplete questionnaires were followed up with phone calls. Consequently, we collected all answers from the questionnaires. In addition, we adopted the dmft index following the recommendation of the WHO [15] as this index is a commonly used index for epidemiological studies in dental research. Thus, the results of the current study can be compared with those of previous studies. However, the study also has some limitations. This analysis was based on cross-sectional data and therefore could only determine associations rather than causal relationships. Furthermore, the information on early life factors was based on parental reports with a long recall period, which might result in greater measurement error. Due to the financial and time constraints, this study used quota sampling, which is a type of non-probability sampling method and may be subject to possible bias. Therefore, the results of the current study cannot represent the caries status of preschool children in Thailand. A longitudinal or cohort study with stratified random sampling should be conducted to determine the associated factors of dental caries in young children in Thailand, where dental caries remains a significant public health issue. 

## 5. Conclusions

In summary, dental caries is highly prevalent in the 4- to 5-year-old Thai children evaluated. Maxillary central incisors had the highest prevalence of caries, whereas mandibular incisors had the lowest prevalence of caries. Caries experience was significantly associated with the child’s age, visible dental plaque, and the mother’s education. Children with increased age, a higher VPI score, and lower mother’s education have a higher chance of having dental caries.

## Figures and Tables

**Table 1:** Prevalence and severity of dental caries in the study children.

Independent Factors	*n*	Caries Free dmft = 0 (*n* = 59)	Caries dmft > 0 (*n* = 249)	Mean dmft ± SD	Mean dt ± SD	Mean mt ± SD	Mean ft ± SD	*p*-Value *
All children	308	59 (19.2%)	249 (80.8%)	8.3 ± 4.7	7.8 ± 4.6	0.5 ± 1.2	<0.1	-
Gender								
male	156	31 (52.5%)	125 (50.2%)	8.4 ± 4.7	8.0 ± 4.6	0.4 ± 1.0	<0.1	0.667
female	152	28 (47.5%)	124 (49.8%)	8.2 ± 4.6	7.6 ± 4.6	0.6 ± 1.4	<0.1	
Age (year)								
4	139	33 (23.7%)	106 (76.3%)	7.9 ± 4.2	7.3 ± 4.0	0.5 ± 1.2	<0.1	0.065
5	169	26 (15.4%)	143 (84.6%)	8.9 ± 5.2	8.5 ± 5.1	0.5 ± 1.2	<0.1	

* *T*-test was adopted to evaluate the association between the child’s sex and age with the dmft score.

**Table 2:** Association between caries experience and children’s demographic background and dental plaque.

Variables	No Caries Experience (dmft = 0) *n* = 59	Caries Experience (dmft > 0) *n* = 249	*p*-Value
Age **	4.9 ± 0.51	5.2 ± 0.5	0.003 *
Gender			
Male	31 (52.5%)	125 (50.2%)	0.746
Female	28 (47.5%)	124 (49.8%)	
Primary care giver			
Father and/or mother	45 (76.3%)	156 (62.7%)	0.048 *
Other relative	14 (23.7%)	93 (37.3%)	
Father’s education			
Mandatory education	30 (50.8%)	147 (59%)	0.253
Higher education	29 (49.2%)	102 (41%)	
Mother’s education			
Mandatory education	19 (32.2%)	117 (47%)	0.040 *
Higher education	40 (67.8%)	132 (53%)	
Family monthly income (THB)			
<10,000	13 (22%)	69 (27.7%)	0.280
10,001–20,000	29 (49.2%)	131 (52.6%)	
>20,001	17 (28.8%)	49 (19.7%)	
Visible plaque index (VPI)			
Low (VPI 0–33%)	4 (6.8%)	3 (1.2%)	0.012 *
Moderate (VPI 33–66%)	22 (37.3%)	73 (29.3%)	
High (VPI 67–100%)	33 (55.9%)	173 (69.5%)	

* *p*-value < 0.05; ** The independent t-test was used for the age variable, whereas the Chi-square test was adopted for all categorical variables.

**Table 3:** Oral health-related behaviors of children with and without caries experience.

	No Caries Experience dmft = 0 (*n* = 59)	Caries Experience dmft > 0 (*n* = 249)	*p*-Value
Age of starting tooth brushing			
1–12 months	26 (44.1%)	75 (30.1%)	0.040 *
Over 12 months	33 (55.9%)	174 (69.9%)	
Assisted tooth brushing			
No	28 (47.5%)	154 (61.8%)	0.043 *
Yes	31 (52.5%)	95 (38.2%)	
Age of starting using toothpaste			
1–12 months	0 (0%)	2 (0.8%)	0.490
Over 12 months	59 (100%)	247 (98.2%)	
Fluoride toothpaste			
Yes	58 (98.3%)	230 (92.4%)	0.096
No	1 (1.7%)	19 (7.6%)	
Tooth brushing frequency			
≤1 time a day	6 (10.2%)	37 (14.9%)	0.350
>1 time a day	53 (89.8%)	212 (85.1%)	
Eating after tooth brushing at nighttime			
No	26 (44.1%)	90 (36.1%)	0.185
Have	33 (55.9%)	159 (63.9%)	
Frequency of snacking			
≤2 times a day	38 (64.4%)	136 (54.6%)	0.173
>2 times a day	21 (35.6%)	113 (45.4%)	

* *p*-value < 0.05.

**Table 4:** Significant caries risk factors in the final model of multiple logistic regression analysis.

Variables	Multivariate	
	Adjusted OR (95%CI)	*p*-Value
Age (year)	2.61 (1.43, 4.75)	0.002
VPIs		
Low (VPI 0–33%)	Reference	1
Moderate (VPI 33–66%)	6.34 (1.23, 32.62)	0.027
High (VPI 67–100%)	10.28 (2.04, 51.75)	0.005
Mother’s education		
Mandatory education	1.99 (1.07, 3.71)	0.031
Higher education	Reference	1

## Data Availability

The data presented in this study are available on request from the corresponding author.
